# Heightened visual attention does not affect inner ear function as measured by otoacoustic emissions

**DOI:** 10.7717/peerj.4199

**Published:** 2017-12-21

**Authors:** W. Wiktor Jedrzejczak, Rafal Milner, Lukasz Olszewski, Henryk Skarzynski

**Affiliations:** 1 Institute of Physiology and Pathology of Hearing, Warsaw, Poland; 2 World Hearing Center, Kajetany, Poland

**Keywords:** Otoacoustic emission, Visual evoked potential, Attention, TEOAE, Medial olivocochlear complex, Contralateral acoustic stimulation, Suppression, Odd-ball

## Abstract

Previous research has indicated that inner ear function might be modulated by visual attention, although the results have not been totally conclusive. Conceivably, modulation of hearing might occur due to stimulation of the cochlea via descending medial olivocochlear (MOC) neurons. The aim of the present study was to test whether increased visual attention caused corresponding changes in inner ear function, which was measured by the strength of otoacoustic emissions (OAEs) recorded from the ear canal in response to a steady train of clicks. To manipulate attention, we asked subjects to attend to, or ignore, visual stimuli delivered according to an odd-ball paradigm. The subjects were presented with two types of visual stimuli: standard and deviant (20% of all stimuli, randomly presented). During a passive part of the experiment, subjects had to just observe a pattern of squares on a computer screen. In an active condition, the subject’s task was to silently count the occasional inverted (deviant) pattern on the screen. At all times, visual evoked potentials (VEPs) were used to objectively gauge the subject’s state of attention, and OAEs in response to clicks (transiently evoked OAEs, TEOAEs) were used to gauge inner ear function. As a test of descending neural activity, TEOAE levels were evaluated with and without contralateral acoustic stimulation (CAS) by broadband noise, a paradigm known to activate the MOC pathway. Our results showed that the recorded VEPs were, as expected, a good measure of visual attention, but even when attention levels changed there was no corresponding change in TEOAE levels. We conclude that visual attention does not significantly affect inner ear function.

## Introduction

The ear is not only passive receiver of sounds but also acts actively, amplifying acoustic signals before transforming them into electrical impulses and sending them to the cerebral cortex. We know this amplification takes place in the cochlea, but the mechanics is complicated and not fully understood (Bell & Fletcher, 2004). However, it is known that efferent neurons to the cochlea play a role in regulating the active process behind the amplification (e.g.: Siegel & Kim, 1982; Veuillet, Collet & Duclaux, 1991). It is possible to study the active process noninvasively by placing a microphone in an ear canal and measuring low-intensity sounds called otoacoustic emissions (OAEs), which are generated as acoustic byproducts of cochlear amplification (Kemp, 1978; Kemp, 2002). Additionally, contralateral acoustic stimulation (CAS) may cause a decrease in cochlear amplifier gain (a decrease in the amplitude of OAEs) via descending medial olivocochlear (MOC) neurons (reviewed by Guinan, 2006). In this way, measurements of OAE strength or strength of their suppression by CAS can become a proxy for the level of activity in neural pathways to the cochlea. Given the link between OAEs and neural activity, the question then arises of whether focusing attention on a sound, or directing attention elsewhere—to another modality such as vision—will, via top-down control, modulate hearing.

In studying the effect of attention on the mechanics of the inner ear, auditory attention has been more frequently used than visual attention. This is probably because auditory attention is obviously more related to functioning of the inner ear. There is quite strong evidence for the effect of auditory attention on OAEs (De Boer & Thornton, 2007; Garinis, Glattke & Cone, 2011; Harkrider & Bowers, 2009; Mishra, 2014; Smith & Cone, 2015). However, it is also of interest to study whether visual attention has an effect on inner ear function, as this may help uncover a relationship between the different modalities—visual and auditory—at the periphery. For example, we are all aware that when we focus intently on something in our visual field we sometimes become oblivious to surrounding sounds. The question we pose in this paper is whether focusing attention on a visual modality might also lead to changes in peripheral parts of the nervous system and express itself as a change in OAEs.

The first study to investigate the effect of visual attention on OAEs (Puel, Bonfils & Pujol, 1988) found that the amplitudes of emissions were reduced during a visual task. Later, attention effects were also shown to enhance the strength with which CAS suppressed OAEs (Froehlich et al., 1990; Ferber-Viart et al., 1995). One of the strongest pieces of evidence for visual attention affecting cochlear function comes from the study by Delano et al. (2007) which showed a decrease in cochlear sensitivity during periods when the subject was attending to visual stimuli (compared to when they were attending to auditory stimuli). A major limitation of this study, however, was that it was done on chinchillas, and used compound action potentials and cochlear microphonics, not OAEs. More recently, a study with human subjects (Wittekindt, Kaiser & Abel, 2014) did find that visual attention had a distinct effect on OAEs, with auditory attention having no discernable effect.

More generally, however, the results are not uniform. There are some contradictory results to those reported above which show no apparent change in amplitude, or suppression of OAEs, during a visual task (Avan & Bonfils, 1992; De Boer & Thornton, 2007). Noteworthy is one study which showed that auditory attention had an effect on OAE amplitudes but no effect was seen when a visual task was employed (De Boer & Thornton, 2007). The results of different experiments show that attention sometimes seems to have an opposite effect on OAEs (e.g., in Smith & Cone, 2015, the effect was to produce higher OAE strength while in Harkrider & Bowers, 2009 the effect was weaker OAEs). In fact, one of the earlier studies of the effect of visual attention on OAEs (Froehlich et al., 1990) found a positive result in only three of 16 subjects, which suggests a possible statistical artefact.

The rationale for this study is to try to settle the issue of the effect of visual attention on auditory function. Here we use objective measures of visual attention to control for its effect and to distinguish it from the effects of auditory attention. In experiments with auditory attention there is always the danger that the stimulus used for evoking attention will also add to the stimulus used to evoke OAEs—that is, a change measured in OAE amplitude might be due to different amounts of acoustic energy delivered to the ear during different auditory tasks (perhaps, for example, due to middle ear muscle effects Guinan et al., 2003). To control attention, most previous studies have relied on instructing the subject to count (or not count) stimuli, and have not controlled attention objectively (Froehlich et al., 1990; Ferber-Viart et al., 1995; De Boer & Thornton, 2007; Walsh, Pasanen & McFadden, 2015). However, it is possible to measure brain activity, and hence attention, by recording evoked potentials. As far as we know, there is only one study of attention effects that has measured evoked potentials while making OAE measurements in humans (Wittekindt, Kaiser & Abel, 2014). However, this study relied on recording steady state potentials and the presence of alpha-wave activity. Such approaches do not provide an ideal measure of attention: steady state potentials might arise from the mere sight of the stimuli, not by attending to them, and although changes in alpha waves might indeed relate to changes in attention (from either being more relaxed or more focused), it is not necessarily the case that such changes are due to the task at hand. To monitor attention more closely, cognitive potentials such as P3 seem more suitable (Rugg & Coles, 1995; Polich, 2007).

The present study uses P3 as an objective measure of visual attention while performing OAE measurements. In this way, it investigates the question of whether attention affects OAE levels in a significant way. By controlling for visual attention, we endeavor to reach a more conclusive finding on OAE amplitudes. As an extra control, we measured OAEs with and without CAS, which as mentioned above, is a known way of affecting OAEs. For this purpose the most common type of OAE used for studies of CAS effect was used, namely transiently evoked OAEs (TEOAEs). To make the result even more definitive, we also introduced another controlling factor into our experiments, one which has not been used before in this context: does simply closing the eyes during OAE measurements—and hence eliminating visual attention entirely—bring about a significant change in OAE amplitudes?

## Material and Methods

### Subjects

Eighteen normally hearing adults (five male, 13 female, age 28–43 years) participated in the study. All subjects had pure tone thresholds better than 25 dB HL at 0.5–8 kHz, normal middle ear function verified by 226 Hz tympanometry (tympanometric peak pressure values between −100 and +100 daPa and peak compensated static acoustic admittance values of 0.2–1.0 mmhos), and no known history of otologic disease. In all subjects, ipsilateral and contralateral middle ear acoustic reflex thresholds for clicks (50 s^−1^) and broadband noise were above 65 dB SPL, i.e., above the levels used in TEOAE measurements.

All subjects had normal or corrected-to-normal vision, and no history of neuropsychiatric diseases or head trauma. They did not take any medications affecting their central nervous system.

All subjects were volunteers and provided written informed consent prior to participation. All procedures were approved by the Ethics Committee of the Institute of Physiology and Pathology of Hearing, Poland (IFPS:KB/09/2015), and conformed to the tenets of the Declaration of Helsinki for medical research involving human subjects.

### Experimental paradigm

The study comprised a basic evaluation of subjects followed by a 30-min recording session divided into three separate parts of about 10 min each: *no task*, *passive*, and *active*, between which the level of attention was varied.

In the *no task* portion of the study, all visual attention was eliminated. The subjects were simply required to sit comfortably in an arm-chair with their eyes closed.

In the *passive* part of the study, subjects just observed visual stimuli presented on a computer monitor 1.5 m in front of them. Psytask 2.84 software (Mitsar Ltd., St Petersburg, Russia) was used to control presentation of the stimuli. Stimuli were delivered according to a visual odd-ball paradigm—a sequence of standard stimuli randomly interrupted by an inverted deviant stimulus). Standard stimuli were gray squares containing a small blue square at the bottom edge; deviant stimuli were the same gray squares but with the small blue square at the top edge. There were 400 (80%) standard stimuli and 100 (20%) deviant stimuli in each recording session. Both standard and deviant stimuli were randomly presented for 100 ms every 2 s. Subjects were asked to just observe the stimuli.

In the *active* part of the session, the same sequence of stimuli was presented but the difference was that the subject was asked to pay attention and silently count the number of deviant stimuli.

During all parts TEOAEs were measured, with and without a contralateral noise suppressor. This meant that during all three parts of a recording session the subject constantly heard click stimuli in one ear and noise, interleaved with silence, in the second ear. During the passive and active parts, visual evoked potentials (VEPs) were acquired.

During some preliminary tests, it was found that sometimes subjects were confused when the order was changed. That is, when the active task was presented first, some would count the stimuli during the passive task, unwittingly repeating the previous test (VEP recordings were similar for active and passive parts). Therefore, in the main experiment the three parts making up a session were always in the same order: *no task*, *passive*, and *active*. As shown later, this resulted in distinctive VEPs for passive and active parts.

All measurements (TEOAEs and VEPs) were performed in a low-noise and electrically shielded room, and details of the procedures are described below.

### TEOAE procedures

OAEs were measured using an ILO 292 system (Otodynamics Ltd., Hatfield, UK). The standard ILO protocol for measuring contralateral suppression of TEOAEs was used: 65 dB SPL clicks (linear mode) were delivered to one ear and 60 dB SPL noise to the contralateral ear (2 s on/off time). All recordings were performed in an acquisition window of 20 ms. To minimize stimulus artifacts the initial part (2.5 ms) of the response was windowed automatically by the system. The main reason for this recording paradigm was to use the default settings of the ILO system as it is likely that such settings will be most often used by other researchers and especially clinicians. The second reason was that TEOAEs evoked by 60 dB are likely to be very weak, and therefore the signal-to-noise ratio (SNR) would be low. Nevertheless, two changes from the default settings of the ILO system were made: the first was to extend the number of averages from 260 to 1,000 (the maximum available); the second was to lower the level of artifact rejection from 47 to 33 dB. These changes were introduced in order to obtain signals with the lowest possible noise floor. Preliminary tests showed that extending the number of averages to 1,000 increased the SNR by about 5 dB and lowered error by a factor of 2 (Jedrzejczak et al., 2017). Measurements of TEOAE level, noise level, and SNR were used for analysis. The requirement was that all recordings should have an SNR of at least 12 dB for recording without CAS, since previous work (Mertes & Goodman, 2016) indicated that high SNR is crucial for reliable TEOAE suppression measurement. In every subject only one ear was tested. The ear with the higher level of TEOAEs was chosen, which resulted in the testing of three left and 15 right ears.

Efforts were also made to search for more local effects. In addition to evaluating suppression for the whole of the signal, suppression was also analyzed in half-octave bands with center frequencies of 1, 1.4, 2, 2.8, and 4 kHz, and in 3.5 ms epochs (cosine window with 0.5 ms rise/fall time and 2.5 ms plateau) between 2.5 and 17.5 ms (the epochs overlapped by 0.5 ms). Since 0.5 ms cosine ramps decayed very fast, the significant part of the signal was located at 2.5 ms plateau fragment. Verifying that the signal existed in particular epochs might be important, since it is known that the few first milliseconds of TEOAEs measured using the linear paradigm often contain artifacts related to reflection of the stimulus from middle ear structures (e.g., Jedrzejczak et al., 2012). Additionally, it is possible that suppression might be stronger during particular epochs or frequency bands (e.g., Smith & Cone, 2015; Jedrzejczak et al., 2016).

### VEP acquisition and analysis

VEPs were used to objectively gauge the subjects’ state of attention during TEOAE recordings. VEPs measurements were based on electroencephalographic (EEG) recordings from a 21-channel system (Mitsar Ltd., St Petersburg, Russia) employing 19 silver chloride electrodes placed in a proprietary EEG cap applied in accordance with the international 10–20 standard (Jasper, 1958). The signal was referenced to electrodes mounted on the ears and digitized at a rate of 250 Hz. The ground electrode was at the FCz position. During EEG acquisition the impedance was monitored and kept below 5 kΩ at all electrodes.

After recording, the EEG signal was analyzed offline using WinEEG 2.84 software (Mitsar Ltd., St. Petersburg, Russia). Digital pre-processing involved high- and low-pass filtering within the range 0.5–15 Hz. Eye-blink artifacts were removed by detecting them using independent component analysis (ICA) and zeroing the activation curves of these individual components (Vigário, 1997; Jung et al., 2000). Epochs with excessive amplitudes (>100 µV overall, or >35 µV in the 20–35-Hz band or >50 µV in the 0–1-Hz band) were excluded from further analysis. Finally, the EEG traces were manually inspected to verify that all artifacts had been properly removed. VEPs were then averaged, relative to stimulus onset, from 200 ms pre-stimulus to 1,000 ms post-stimulus.

The P3 wave was defined as the largest positive wave in the 250–600 ms window, with amplitude measured in the parietal electrodes (P3, Pz, and P4) and only in VEPs recorded in response to deviant stimuli.

### Analysis

In all analyses, a 95% confidence level (*p* < 0.05) was chosen as the criterion of significance. For all parameters the statistical significance of the mean difference between groups was evaluated by repeated measures analysis of variance (RM ANOVA). For multiple comparisons, Bonferroni corrections were applied. All datasets were checked for sphericity by Mauchly’s test. Greenhouse–Geisser correction for violation of sphericity was applied when needed. Percent data were transformed to a normal distribution by applying an arcsine transformation.

## Results

### VEPs

**Figure 1:**
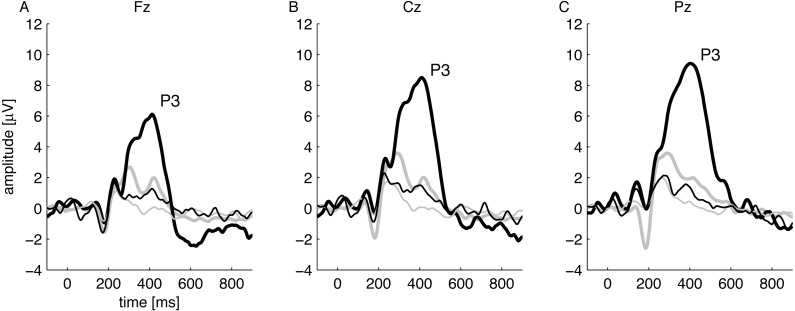
Grand-average VEPs for all subjects recorded during passive (thin lines) and active (thick lines) conditions using standard stimuli (grey lines) and deviant stimuli (black lines). The consecutive (A), (B), and (C) show recordings from Fz, Cz, and Pz electrodes (10/20 standard), respectively. P3 only appears in response to odd-ball stimuli.

Attention states during passive and active parts of the experiment were verified objectively by VEP analysis. That is, it was verified that in each subject, in response to the instruction to pay attention and count the deviant stimuli, the P3 wave—an electrophysiological marker of cognition (Rugg & Coles, 1995; Polich, 2007)—was present during the active part but absent during the passive part. Figure 1 shows, for three electrodes, the grand-average VEPs for all subjects during both passive and active periods, and it is clear that a large P3 wave appears only when the subject was counting deviant stimuli (thick black lines in figure).

### TEOAE parameters

Since the main interest was to gauge the effect of attention on the strength of suppression of TEOAEs, it was crucial to verify that basic TEOAE parameters stayed the same over the three different conditions (no task, passive, and active). The parameters typically used for TEOAE analysis are response level, noise level, and SNR.

**Figure 2:**
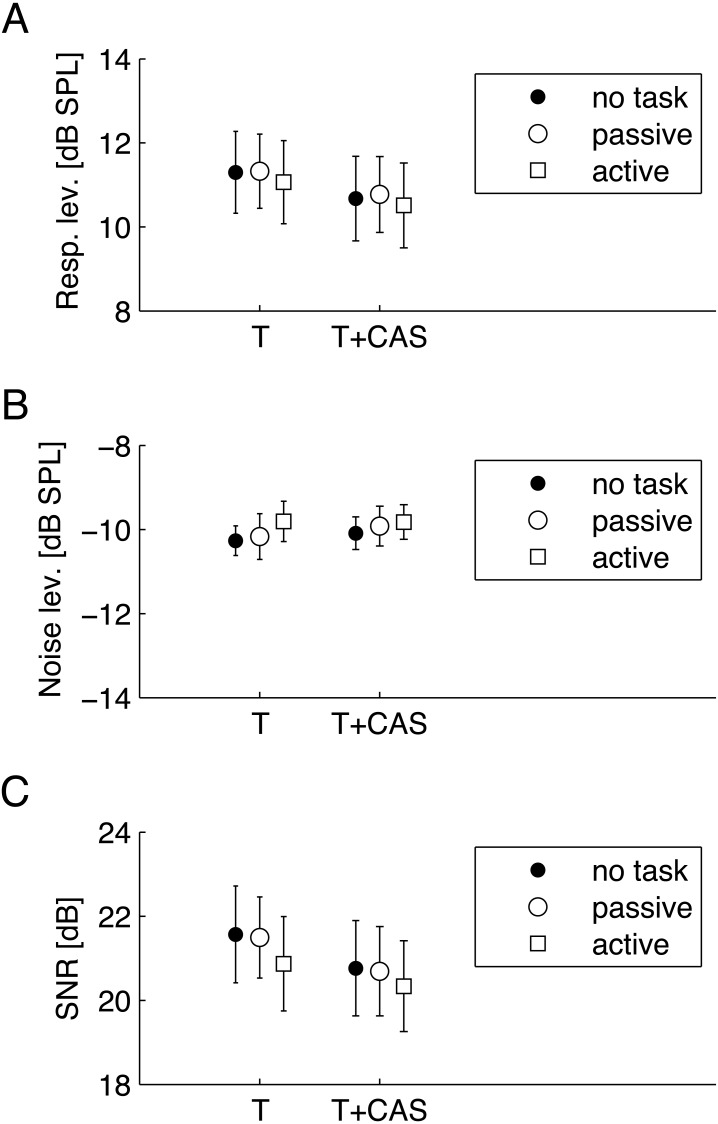
Average TEOAE parameters for three conditions—no task, passive visual, and active visual—for TEOAEs measured without (T) and with (T+CAS) contralateral stimulation by noise. (A) response levels; (B) noise levels; (C) signal-to-noise ratio (SNR). Whiskers indicate standard errors. There were no statistically significant differences between different attention conditions (*p* > 0.05). Response levels and SNRs without CAS (T) were significantly greater than with CAS (T+CAS).

Figure 2 shows average levels of these parameters. They were calculated for TEOAEs measured without CAS (marked as T) and with CAS (marked as T+CAS). A two-factor RM-ANOVA was conducted for each parameter as a function of CAS (T/T+CAS) and task (no task/passive/active). For response level, a statistically significant main effect of CAS was found (*F*[1, 17] = 24.43, *p* < 0.001, ${\eta }_{p}^{2}=0.59$), while the main effect of task (*F*[1.18, 20.07] = 0.14, *p* = 0.75, ${\eta }_{p}^{2}=0.008$), or an interaction of CAS and task (*F*[1.42, 24.14] = 1.65, *p* = 0.215, ${\eta }_{p}^{2}=0.088$), were not significant. For noise level, no effect was significant (main effect of task: *F*[2, 34] = 0.52, *p* = 0.602, ${\eta }_{p}^{2}=0.029$; main effect of CAS: *F*[1, 17] = 1.62, *p* = 0.22, ${\eta }_{p}^{2}$ = 0.087; interaction effect of CAS and task: *F*[2, 34] = 0.21, *p* = 0.811, ${\eta }_{p}^{2}=0.012$). Similarly to response level, for SNR only the main effect of CAS was significant (*F*[1, 17] = 15.07, *p* = 0.001, ${\eta }_{p}^{2}=0.47$), while the main effect of task (*F*[2, 34] = 0.43, *p* = 0.656, ${\eta }_{p}^{2}=0.025$), or an interaction of CAS and task (*F*[2, 34] = 0.26, *p* = 0.776, ${\eta }_{p}^{2}=0.015$), were not significant.

### TEOAE suppression

**Figure 3:**
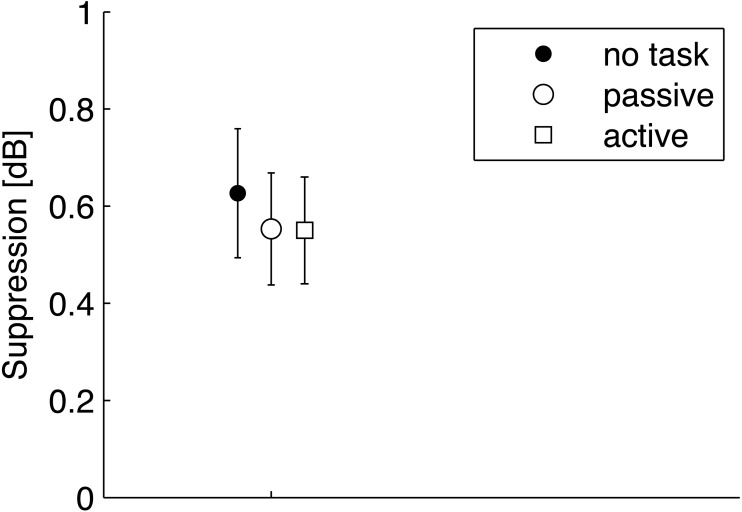
Average suppression levels for TEOAEs measured in the no task, passive, and active visual attention conditions. Whiskers indicate standard errors. There were no statistically significant differences between any of the data (*p* > 0.05).

The magnitude of TEOAE suppression due to CAS was calculated by subtracting TEOAE response levels measured with CAS (shown at the Fig. 2A as T+CAS) from those measured without CAS (marked with T). The suppression levels were small, on average around 0.6 dB, and are plotted in Fig. 3. The average suppression levels for passive and active attention conditions were very similar, with slightly higher suppression levels for the no task condition. However, RM ANOVA as a function of task did not yield a significant result (*F*[1.42, 24.14] = 1.65. *p* = 0.215, ${\eta }_{p}^{2}=0.088$).

**Figure 4:**
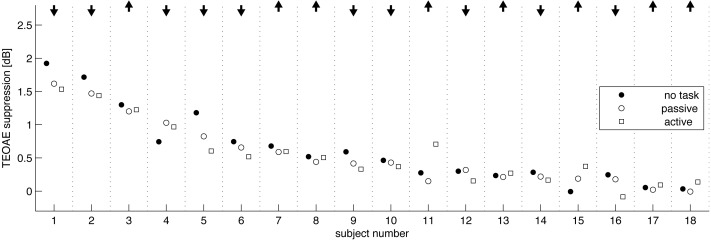
Amount of TEOAE suppression by individual subject, and shown in order of average suppression. Arrows at top indicate whether, suppression was raised (up arrow) or lowered (down arrow) in the active condition compared to the passive condition. In 10 subjects suppression was lower during the active condition and in 8 it was higher.

When responses from single subjects were investigated (Fig. 4), it was possible to investigate individual changes in OAEs between attention states—for example, there might be situations where the change in OAEs between attention states for one subject was in a different direction to that for another, i.e., in one person OAEs might be enhanced while diminished in a second. Here, in 10 subjects suppression was lower during the active condition compared to the passive condition, and in eight it was higher. As these are nearly equal numbers, there does not seem to be a trend.

### Additional factors

**Figure 5:**
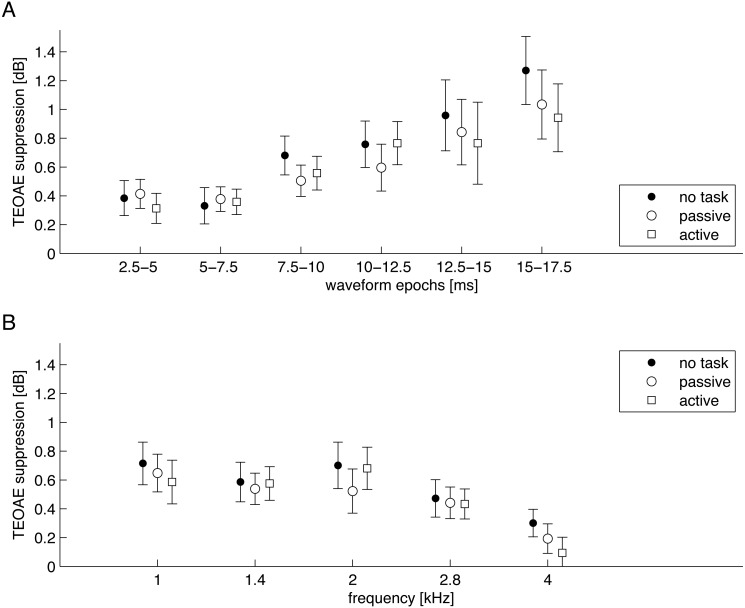
Average suppression levels for TEOAEs measured in the three conditions shown for different epochs of the signal (A) and different half-octave frequency bands (B). Whiskers indicate standard errors. There were no statistically significant differences between different attention conditions (*p* > 0.05).

In order to investigate if the sought-after effects were more specific, the signals were split into separate frequency bands and different time windows. First the signals were separated into 2.5 ms epochs (Fig. 5A). A two-factor RM-ANOVA was conducted as a function of epoch and task. A statistically significant main effect of epoch was found (*F*[2.13, 36.12] = 8.56, *p* = 0.001, ${\eta }_{p}^{2}=0.335$), while the main effect of task (*F*[1.48, 25.18] = 2.76, *p* = 0.096, ${\eta }_{p}^{2}=0.140$) or interaction between epoch and task (*F*[4.30, 73.1] = 1.05, *p* = 0.392, ${\eta }_{p}^{2}=0.058$) were not significant. Second, the signals were split into half-octave frequency bands at 1, 1.4, 2, 2.8, and 4 kHz (Fig. 5B). A two-factor RM-ANOVA was conducted as a function of frequency and task. Similarly, a statistically significant main effect of frequency was found (*F*[4, 68] = 7.18, *p* < 0.001, ${\eta }_{p}^{2}=0.297$), while the main effect of task (*F*[2, 34] = 2.50, *p* = 0.097, ${\eta }_{p}^{2}=0.128$) or interaction between frequency and task (*F*[4.71, 80.06] = 1.03, *p* = 0.406, ${\eta }_{p}^{2}=0.057$) were not significant.

Since the average suppression was small (around 0.6 dB), analysis was focused on the subgroup that showed suppression >0.5 dB under all conditions—a total of seven ears. However, even here the results did not change. There was still no significant change in suppression between states of attention and inattention.

**Figure 6:**
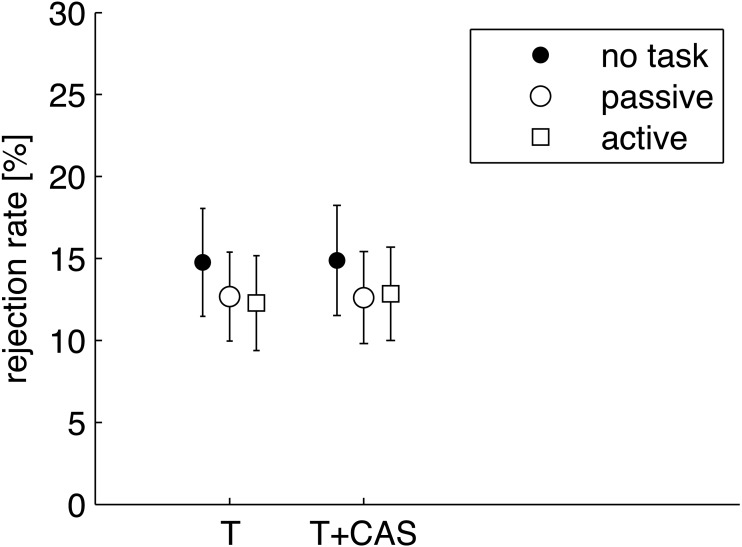
Average rejection rate of noisy trials of TEOAEs. The parameters were calculated for TEOAEs signals measured without (T) and with contralateral stimulation by noise (T+CAS). Whiskers indicate standard errors. There were no statistically significant differences between any of the data (*p* > 0.05).

Finally, circumstances surrounding the rejection rate for each condition were investigated. The initial measurement procedure had been done in such a way that responses which exceeded a certain level (here a level of 33 dB) were rejected. The rejected responses are taken to be signals with excess noise related to effects such as body movements, swallowing, or loud breathing, but they can also relate to body tension, and this might relate directly to states of attention. The rejection rate was transformed into a percentage of responses rejected, and figures of 12–17% were returned (Fig. 6). The rate was similar under each condition. A two-factor RM-ANOVA as a function of CAS (T/T+CAS) and task (no task/passive/active) did not show any significant effects (main effect of task: *F*[1.51, 25.59] = 0.44, *p* = 0.649, ${\eta }_{p}^{2}=0.025$; main effect of CAS: *F*[1, 17] = 0.55, *p* = 0.468, ${\eta }_{p}^{2}=0.031$; interaction effect of CAS and task: *F*[2, 34] = 1.50, *p* = 0.238, ${\eta }_{p}^{2}=0.081$).

## Discussion

To date, the evidence for an effect of visual attention on OAEs has seemed ambiguous, as several papers have reported contradictory results. Therefore, in the present study, great care was taken into designing an experiment in such a way that attention states could be objectively confirmed by VEPs. At the same time, we wanted to achieve the best quality OAEs. In most prior studies the attention state was not monitored with evoked potentials, so in fact the authors could only assume—based on an instruction to count stimuli—that the attention states were different (e.g., Froehlich et al., 1990; Meric & Collet, 1992; Ferber-Viart et al., 1995; De Boer & Thornton, 2007; Walsh, Pasanen & McFadden, 2015). Here, visual attention was objectively confirmed by the presence of a P3 wave, which was present during active counting and absent during passive conditions (Fig. 1).

The present study has shown no significant effect of visual attention on TEOAEs, even though we measured, objectively, different patterns of VEPs arising from each attention state. There were no significant differences between response levels of TEOAEs and levels of suppression of TEOAEs. In addition, although some previous studies have seen an increase in suppression during active attention (Ferber-Viart et al., 1995), here only eight out of 18 subjects showed an increase during the active attention task. Finally, there were also no significant difference between TEOAEs measured during visual tasks and when eyes were closed (the no task condition).

Our results contradict those of previous studies which have found that visual attention has an effect on TEOAEs (Froehlich et al., 1990; Meric & Collet, 1992; Ferber-Viart et al., 1995). However, even among the three positive findings cited, the effect seems to be weak. For example, Froehlich et al. (1990) found only three of their 16 subjects showed a clear effect of visual attention. The present study in fact supports more recent work (De Boer & Thornton, 2007) which also did not find any influence of visual attention, and two earlier studies (Avan & Bonfils, 1992; Michie et al., 1996).

The main uncertainty surrounding most previous studies is that the authors did not describe if or how they controlled SNR. As shown by Goodman et al. (2013), high SNRs (above 9 dB) are needed in order to minimize the inter-measurement variability of small TEOAE suppression effects and achieve a definite result. Here it has been shown that even with a considerable SNR value (22 dB on average) there seems to be no attention effect. In our experience with the ILO system (e.g., Jedrzejczak et al., 2017), it is rare to achieve similar SNRs using the default number of averages (260), as typically used in previous studies.

The levels of contralateral suppression measured in the present study were similar to those recorded in previous studies using the same equipment (e.g., Stuart & Cobb, 2015; Killan et al., 2017). It should be noted, however, that the levels of click stimuli used to evoke TEOAEs and the levels of contralateral noise in those studies varied slightly from the levels used in the present work. Also the changes of the parameters of TEOAE suppression for different signal epochs and frequency bands were similar to those in previous studies, i.e., suppression increased in later epochs (similarly to Smith & Cone, 2015), and diminished with higher frequency (similar to Jedrzejczak et al., 2016).

Two previous studies (De Boer & Thornton, 2007; Walsh, Pasanen & McFadden, 2014) have pointed out that the number of trials rejected because of excessive noise might be related to different attention states. However this was not the case here. It should be mentioned that we asked our subjects to count silently and not make any moves during recording, whereas in some other studies counting was achieved by tapping a button (De Boer & Thornton, 2007; Walsh, Pasanen & McFadden, 2014; Wittekindt, Kaiser & Abel, 2014). Certain studies (Garinis, Glattke & Cone, 2011; Kalaiah et al., 2017; Smith & Cone, 2015) suggested that the effect of attention might be more pronounced in particular parts of the signal. We pursued this avenue but found that attention effects did not arise either in particular time epochs or frequency bands.

The present results suggest there does not need to be any control for attention during MOC measurements of OAEs, as has been suggested by Mertes & Goodman (2016). This is especially true in typical measurement situations where there are smaller number of averages, lower SNR, and higher variability.

Our results lead us to think that the effect of visual attention on TEOAEs does not exist. Apart from this negative conclusion, what other explanation might there be for our findings? It might be argued that the effect exists, but that the current procedure is not sensitive enough to detect it. However we reiterate that very strict criteria were used (SNR >12 dB), much higher than in standard measurements (e.g., SNR >3 or >6 dB, Mishra & Lutman, 2013; Lisowska et al., 2014; Micarelli et al., 2016; Killan et al., 2017). Another reason for seeing no effect might be because all TEOAE parameters, and especially contralateral suppression effects on TEOAEs, have high variability, even when refitting of the probe is avoided (Mishra & Lutman, 2013; Stuart & Cobb, 2015; Jedrzejczak et al., 2016). In the same vein, previous studies by other teams (Marshall et al., 2014; Mertes & Goodman, 2016; Killan et al., 2017) have also found that suppression varies greatly from one subject to another, suggesting that effects can only be seen in particular subjects. The general conclusion of these recent studies was that TEOAE measurements with CAS might be not sufficiently repeatable to detect subtle changes in MOC activity (Mertes & Goodman, 2016; Killan et al., 2017). The case might also be made that the effect is more pronounced with other types of OAEs—e.g., distortion product OAEs (DPOAEs) (Wittekindt, Kaiser & Abel, 2014; Srinivasan et al., 2014) or stimulus frequency OAEs (SFOAEs) (Walsh, Pasanen & McFadden, 2015). We leave it to others to explore these slight possibilities.

There is also some work (Delano et al., 2007) that suggests that the effect of visual attention is stronger for higher attentional demand tasks, from which it might be inferred that the task used here was too easy. Countering this suggestion, the visual task we chose was one that was able to generate robust VEPs. There has been one study where a more complicated visual task was chosen (Srinivasan et al., 2014), and indeed this study found that visual attention had an effect on DPOAEs; unfortunately, however, there was no monitoring of the level of attention using evoked potentials.

Finally, the limitations of this study should be mentioned. The group studied was quite small (18 subjects), although it was similar in size to those in which an effect of attention was detected (Froehlich et al., 1990; Meric & Collet, 1992; Garinis, Glattke & Cone, 2011; Harkrider & Bowers, 2009; Smith & Cone, 2015; Wittekindt, Kaiser & Abel, 2014). There is also the possibility of the middle-ear muscle reflex confounding the results, as it is possible to activate the reflex in some subjects even with a 60 dB SPL stimulus (e.g.: Guinan et al., 2003; Marks & Siegel, 2017).

It is also possible that tiredness had an effect. To reduce this factor to a minimum, we tried to keep the experiment as simple as possible. Adding some other requirements to arouse attention (e.g., attending to clicks or contralateral stimuli) might allow our results to be strengthened, but some of our subjects reported that the test was quite tiring as it was.

It would also be interesting to compare visual and auditory attention within the same paradigm. However it is not easy to do in terms of P3 alone. One solution might be to embed stimuli within the click train or the contralateral noise which need to be attended to (a technique similar to that used by De Boer & Thornton, 2007). In the present work, however, the aim was to use as simple a paradigm as possible and not introduce too many additional factors.

## Conclusions

The present study indicates that there seems to be no effect of visual attention on the level of suppression of TEOAEs. There are several possible explanations: (1) There is no effect at all; (2) there is an effect but it manifests only under more demanding conditions (i.e., the present task was too easy); (3) the effect is present under the conditions used but it is smaller than the reliability of measurements.

In short, we cannot definitely say that there is no effect of visual attention on TEOAEs and the effect of their contralateral suppression. It is impossible to prove a negative, and all we can conclude is that the effect is simply not measurable under current conditions. The present study, along with other recent studies, showed quite high variability in TEOAE contralateral suppression, a factor which makes detecting small effects very difficult. Nevertheless, we can say there seems to be no need to control visual attention during standard TEOAE tests or TEOAE suppression tests. The main TEOAE parameters seem to be unaffected, even with eyes closed. An alternative way of saying the same thing is that experimenters should be free to use any visual task they like while recording TEOAEs (if, for example, it is necessary to keep the subject awake during long periods of testing) as there is little risk that visual stimuli will influence the results.

##  Supplemental Information

10.7717/peerj.4199/supp-1 Supplemental Information 1 VEP and OAE data.

## Additional Information and Declarations

### Data Availability

The following information was supplied regarding data availability: The raw data has been provided as a Supplemental File.
